# The Clustering Analysis of Time Properties in Patients With Cerebral Small Vessel Disease: A Dynamic Connectivity Study

**DOI:** 10.3389/fneur.2022.913241

**Published:** 2022-06-20

**Authors:** Wenwen Yin, Xia Zhou, Chenchen Li, Mengzhe You, Ke Wan, Wei Zhang, Wenhao Zhu, Mingxu Li, Xiaoqun Zhu, Yinfeng Qian, Zhongwu Sun

**Affiliations:** ^1^Department of Neurology, The First Affiliated Hospital of Anhui Medical University, Hefei, China; ^2^Department of Radiology, The First Affiliated Hospital of Anhui Medical University, Hefei, China

**Keywords:** cerebral small vessel disease, dynamic functional connectivity, sliding window, clustering analysis, cognitive impairment

## Abstract

**Purpose:**

This study aimed to investigate the dynamic functional connectivity (DFC) pattern in cerebral small vessel disease (CSVD) and explore the relationships between DFC temporal properties and cognitive impairment in CSVD.

**Methods:**

Functional data were collected from 67 CSVD patients, including 35 patients with subcortical vascular cognitive impairment (SVCI) and 32 cognitively unimpaired (CU) patients, as well as 35 healthy controls (HCs). The DFC properties were estimated by k*-*means clustering analysis. DFC strength analysis was used to explore the regional functional alterations between CSVD patients and HCs. Correlation analysis was used for DFC properties with cognition and SVD scores, respectively.

**Results:**

The DFC analysis showed three distinct connectivity states (state I: sparsely connected, state II: strongly connected, state III: intermediate pattern). Compared to HCs, CSVD patients exhibited an increased proportion in state I and decreased proportion in state II. Besides, CSVD patients dwelled longer in state I while dwelled shorter in state II. CSVD subgroup analyses showed that state I frequently occurred and dwelled longer in SVCI compared with CSVD-CU. Also, the internetwork (frontal-parietal lobe, frontal-occipital lobe) and intranetwork (frontal lobe, occipital lobe) functional activities were obviously decreased in CSVD. Furthermore, the fractional windows and mean dwell time (MDT) in state I were negatively correlated with cognition in CSVD but opposite to cognition in state II.

**Conclusion:**

Patients with CSVD accounted for a higher proportion and dwelled longer mean time in the sparsely connected state, while presented lower proportion and shorter mean dwell time in the strongly connected state, which was more prominent in SVCI. The changes in the DFC are associated with altered cognition in CSVD. This study provides a better explanation of the potential mechanism of CSVD patients with cognitive impairment from the perspective of DFC.

## Introduction

Cerebral small vessel disease (CSVD) is a common neurological disease in elderly individuals and is clinically characterized by progressive cognitive impairment, especially executive dysfunction (1). The mechanism of CSVD and the consequential cognitive impairment have been explored from various perspectives (2, 3). Notably, previous studies have tried to explore the underlying pathogenesis of CSVD from the perspective of the brain functional network, indicating that CSVD is a “disconnection syndrome” (3), with the characteristics of extensive structural and functional connectivity disruption in large-scale brain networks, such as the default mode (4–6), dorsal attention (5), frontoparietal control (6) and salience networks (7). Most of these studies were based on resting-state functional MRI (rs-fMRI), which reflects the spontaneous activity of the brain (7). Rs-fMRI has been consistently considered a promising measurement to investigate brain connectivity alterations in both the resting state and during the execution of specific tasks. Nevertheless, increasing evidence has shown that the functional activity of the brain in the resting state is not static but constantly dynamic during scanning (8, 9), and the dynamic fluctuations of brain connectivity analysis could reveal more information that cannot be observed in the static state.

Dynamic functional connectivity (DFC) analysis is widely utilized to explore the time-varying profiles of functional connectivity and has been used in the study of several neurodegenerative diseases (10–17). For example, Fu et al. presented that language, attention, and processing speeds were negatively correlated with weakly connected states and positively correlated with strongly connected states in Alzheimer's disease (AD) patients (10). Moreover, the temporal properties and spatial pattern of the cognitive control network were suggested to serve as biomarkers that could differentiate the stages of AD (11). The changes in DFC strength in different frequency bands were reported to be related to both positive and negative symptoms in patients with schizophrenia (12, 13). The DFC states were also suggested to be associated with cognitive decline in Parkinson's disease, specifically, increased mean dwell time (MDT) in the segregated state and reduced number of transitions between states are associated with dementia in Parkinson's disease (16, 17). These studies presented the valuable roles of DFC analysis in elucidating the mechanisms underlying cognition and mental symptoms. However, few studies have focused on CSVD from the perspective of DFC. A recent functional network study showed that patients with subcortical ischaemic vascular disease (SIVD), which resembles the disease of CSVD, dwelled longer in the weakly connected state, which may be a feature of SIVD (10). However, whether patients with subcortical vascular cognitive impairment (SVCI) and cognitively unimpaired CSVD (CSVD-CU) exhibit similar DFC changes and whether the altered DFC is associated with cognitive impairment in CSVD patients or can be seemed as a biomarker remain unclear.

Herein, the differences in dynamic connectivity between healthy controls (HCs) and patients with CSVD were systematically investigated and the relationships between dynamic connectivity changes with cognitive impairment were also explored. The CSVD patients were hypothesized with a higher proportion and longer MDT in the sparsely connected state, while a lower proportion and shorter MDT in the strongly connected state in CSVD patients, which may be correlated with cognitive impairment.

## Materials and Methods

### Subjects

This cross-sectional study included 67 consecutive CSVD patients recruited from the First Affiliated Hospital of Anhui Medical University between July 2018 and July 2021, with 35 HCs (patient's spouse or other family members) matched by age, gender, and education. The general information, comorbidities, and histories of smoking and drinking were collected from all participants.

The inclusion criteria for CSVD patients were as follows: (a) aged 50–80; (b) presented at least one common symptom of CSVD, including cognitive decline, gait, and balance disorders, mood or sleep disorders, and urinary and stool disorders; or a history of symptomatic stroke (lacunar stroke syndrome, symptoms lasting more than 24 h and occurring more than 6 months before visiting; or transient ischaemic attack lasting <24 h, with limb weakness, semisensory loss or dyskinesia in the preceding 6 months); and (c) met any of the following brain MRI marker criteria for CSVD, such as white matter hyperintensities (WMH), lacunar infarction (LI), and enlarged perivascular spaces (EPVS) using daily routine brain MRI scan (18, 19). The WMH was defined as the hyperintensities on fluid-attenuated inversion-recovery (FLAIR) and was further graded according to the Fazekas scale (20). The Fazekas score was calculated as the sum of periventricular and deep WMH, ranging from 0 to 6. The severity of WMH was categorized as follows: none to mild WMH (Fazekas scale score 0–2) and moderate to severe WMH (Fazekas scale score 3–6) (18); LI was defined as round or ovoid fluid-filled cavities of 3–15 mm on T2 weight and FLAIR images (21). EPVS was defined as small (3 mm) punctate (if perpendicular) and linear (if longitudinal to the plane of scan) hyperintensities on T2 images in the basal ganglia or centrum semiovale, and they were rated on a previously described, validated semiquantitative scale from 0 to 4. The severity of EPVS was categorized as follows: none to mild (grade 0–1) and moderate to severe (grade 2–4) (21).

Then, CSVD with subcortical vascular cognitive impairment (SVCI) was defined as having either mild cognitive impairment or dementia (22) according to impairment in MMSE as follows: MMSE scores were based on the following educational levels of Chinese people: illiteracy ≤ 19, primary school ≤ 22, junior middle school, and above ≤ 26 (23).

The exclusion criteria were as follows: (a) patients with brain tumors or other systemic malignant tumors; (b) history of brain trauma; (c) large area of new infarction with a diameter > 2 cm; (d) dysfunction of liver, kidney, heart, lung, or other vital organs; (e) history of craniocerebral surgery; (f) CT or other imaging examinations of cerebral hemorrhage; (g) acute ischaemic stroke caused by atrial fibrillation and cardiogenic embolism; (h) other CSVD etiologies secondary to heredity, infection, autoimmune inflammation, poisoning, radiation, metabolic encephalopathy, and sporadic cerebral amyloid vascular disease; (i) history of psychiatry disease or history of using psychiatry drugs within the past 3 months; and (g) history of diseases that may affect cognition, such as Parkinson's disease, dementia with Lewy bodies, frontotemporal dementia, and AD. The exclusion and inclusion processes of participants are shown in Supplementary Figure S1.

The present study was approved by the First Affiliated Hospital of Anhui Medical University Subcommittee on Human Studies (Ethics ref. Quick-PJ2021-15-33). Written informed consent was obtained from all study subjects after a full explanation of the procedure.

### Total MRI Burden of CSVD

According to the previous study (24), the total MRI burden of SVD was rated from 0 to 4 by counting the presence of 4 traditional MRI features of SVD, including WMH (1 point), LI (1 point), cerebral microbleed (CMB) (1 point) and EPVS (1 point). However, the CMB had not been evaluated in our study due to the lack of susceptibility weighted imaging (SWI) sequences (19). Therefore, the total scores of SVD in our study were 3. The presence of WMH was defined as periventricular Fazekas score = 3 or subcortical Fazekas score ≥ 2 (1 point if present). The presence of LI was defined as the presence of one or more lacunes (1 point if present). The presence of EPVS was counted as 1 point if there were moderate to severe (grade 2–4) EPVS in the basal ganglia.

### Neuropsychological Assessment

Cognition assessment of both CSVD patients and HCs was performed by two trained neuropsychological technicians within 1 week after the MRI scan. All participants were evaluated by the Mini-Mental State Examination (MMSE) (25), Montreal Cognitive Assessment (MoCA) (26), Cambridge Cognitive Examination-Chinese version (CAMCOG-C) (27–29), and Activities of Daily Living (ADL) scales (30). The impairments in specific cognition domains were further extracted by the subscores of CAMCOG-C. Moreover, executive function was further assessed using the Stoop Color-Word Test (31) (SCWT-A dot, SCWT-B words, SCWT-C dot-word).

### Image Acquisition

All images of participants were collected on a 3.0 Tesla GE Signa HDxt MRI scanner (GE, Milwaukee, WI, USA) with an 8-channel head coil. During the scanning, the participants were instructed to remain quiet, close their eyes, and not think of anything as much as possible. The scanning sequences included high-resolution 3D T1-weighted (3D-T1) structural images, T2 FLAIR images, and resting-state blood oxygen level-dependent (BOLD) fMRI. The 3D-T1 structural images were acquired by employing a spoiled gradient recalled echo sequence with the following parameters: repetition time (TR) = 9.5 ms; echo time (TE) = 3.9 ms; flip angle (FA) = 20°; field of view (FOV) = 256 mm × 256 mm; matrix size = 512 × 512; slice thickness = 1 mm. The parameters of T2 FLAIR images were as follows: TR = 11 s; TE =120 ms; FA = 90; 22 contiguous, 5-mm-thick axial slices; matrix size = 512 × 512; and FOV = 230 × 230 mm^2^. Functional MRI images were collected using echo-planar imaging (EPI) at 2-s interval sequences with the following parameters: TR = 2,000 ms; TE = 30 ms; flip angle = 80°; FOV = 240 mm × 240 mm; matrix size = 64 × 64; slice thickness = 4 mm; and slice ga*p* = 0.6 mm. The total BOLD duration was 8 min, with a total of 240 time points.

### Data Preprocessing

The rs-fMRI data were preprocessed by Data Processing and Analysis for Brain Imaging (http://rfmri.org/dpabi) (32). The following preprocessing steps were taken: (a) the first 10 volumes were removed to allow the signal to reach equilibrium, and the remaining 230 time points were then processed; (b) the remaining volumes were corrected for acquisition time delay between slices; (c) all volumes were then realigned to the mean volume to correct for head motion; (d) functional images were spatially normalized to the Montreal Neurological Institute space with resampled voxel size = 3 × 3 × 3 mm^3^; (e) detrending was performed to remove the linear trends; (f) temporal bandpass filtering (0.01–0.08 Hz) was used to reduce high-frequency physiology noise and low frequency drift; and (g) white matter, cerebrospinal fluid, and head motion nuisance variables were removed using a Friston 24-parameter model. Participants were excluded if their head motion parameters exceeded 2.5 mm translation and/or 2.5° rotation. The mean framewise displacement (FD) value was calculated by averaging the FD of each subject across the time points, and no significant differences were found between CSVD patients and HCs (*p* = 0.887). Furthermore, the mean FD value was calculated as an additional motion correction.

### Construction of Dynamic Functional Connectivity

The dynamic brain connectome analysis toolbox (http://restfmri.net/forum/ DynamicBC) was applied to compute the DFC. The average time courses were extracted from 90 regions of interest (ROIs) of Anatomical Automatic Labeling atlas (AAL) (excluding the cerebellum) to calculate the functional connectivity, and the widely used sliding window approach was adopted to calculate DFC alterations, with a width of 50 TR (100 s) slides in steps of 1 TR (2 s) according to previous studies. Then, the functional connectivity in each window was calculated, resulting in a time series of functional connectivity matrices (90 × 90) for the next analysis. The average DFC strength was calculated as follows:


$$\begin{array}{l} \text{DFC}-\text{Strength}\left(i,j\right)=\frac{1}{T}\overset{T}{\underset{t=1}{\sum }}Z{\left(i,j\right)}_{t} \end{array}$$

where *Z*(*i, j*)_*t*_ is the value of functional connectivity (Fisher-z transformed) between ROI i and ROI j in time window t, and T is the number of windows (33).

### Clustering Analysis

K-means clustering was applied to estimate the reoccurring functional connectivity states over time using the squared Euclidean distance. The optimal number of clusters estimated using the elbow criterion was 3 (*k* = 3), which was computed as the ratio between the within-cluster distance and between-cluster distances (34). Three temporal properties of the DFC states were evaluated, the fractional windows (proportion of total time window for each state in all participants), MDT (the time the subject stayed in the specific state before changing to another state), and the total number of transitions (the number of changes between the 3 connectivity states).

### Validation Analysis

To validate the robustness of the temporal properties, different window sizes (20 TRs, 30 TRs, 40 TRs and 60 TRs) and a range of cluster sizes were applied to retest the results (*k* = 2, 4, 5 and 6).

### Blinding

All assessments were performed by two trained neurologists who were blinded to the subjects' diagnoses. When the two neurologists had questions or disputes about the subject's cognitive function scores or imaging classification, a third experienced neurology expert was invited to make the final decision.

### Statistical Analysis

Two-sample *t-*tests and χ^2^ tests were used to compare demographic data between the CSVD and HC groups with SPSS version 23.0. A general linear model (GLM) was used to evaluate between-group differences of all metrics extracted from DFC (fractional windows, MDT, number of transitions) with age, gender, education, and mean FD as covariates (Bonferroni correction, *P* < 0.05). To identify the altered DFC strength between CSVD patients and HCs, two-sample t-tests with age, gender, education, and mean FD as covariates were performed in GRETNA (http://www.nitrc.org/projects/Gretna) (35) with network-based statistics (NBS) correction (edge *P* < 0.001, component *P* < 0.05, 1,000 iterations). Partial correlation analysis was conducted between DFC temporal properties and cognition scales in CSVD patients with age, gender, education, and mean FD as covariates (*P* < 0.05). The Spearman's correlation was conducted between SVD scores and temporal properties (*P* < 0.05).

## Results

### Demographics, Clinical and Cognitive Characteristics

The participants' demographic and neuroimaging characteristics are provided in Table 1. For the neuropsychological scale, the general cognitive function of the CSVD patients was worse than that of the HCs, indicated by decreased MMSE and COMCOG-C scores, especially in the SVCI subgroup. In the CAMCOG-C subitems, CSVD patients showed decreased language, memory, praxis, calculation, abstraction, and perception, indicating extensive cognitive impairment. Furthermore, executive dysfunction was found in the CSVD group, as indicated by their worse performance on the SCWT. CSVD scores were presented in Table 2. The SVD scores of 35 HCs were 0, 46 patients with CSVD were scored 1, 17 patients were scored 2, and 4 patients were scored 3.

**Table 1:** Demographic and clinical data of the CSVD and HC groups.

	**HC (N=35)**	**CSVD (N=67)**	**t/χ2**	***P* value**	**Cohen's d/Φ**
Age, years	64.89 ± 7.80	67.51 ± 7.52	−1.65	0.102^a^	−0.344
Female, *n* (%)	19(54%)	31(46%)	0.591	0.442^b^	0.076
Education, years	9.19 ± 4.49	9.12 ± 3.67	0.08	0.936^a^	0.017
BMI, kg/m^2^	23.46 ± 2.51	24.61 ± 2.83	−2.025	0.045^a^	−0.422
Alcohol abuse, *n* (%)	9(26%)	13(19%)	0.541	0.462^b^	−0.073
Current smoking, *n* (%)	6(17%)	16(24%)	0.617	0.432^b^	0.078
Commorbidities					
Hypertension, *n* (%)	13(37%)	30(45%)	0.549	0.459^b^	0.073
Diabetes mellitus, *n* (%)	2(6%)	9(13%)	0.734	0.391^b^	0.118
Dyslipidaemia, *n* (%)	5(14%)	11(16%)	0.079	0.779^b^	0.028
CAD, *n* (%)	2(6%)	11(16%)	1.504	0.22^b^	0.152
MMSE	27.71 ± 1.64	25.75 ± 2.90	3.713	<0.001^a^	0.774
MoCA	24.06 ± 3.23	21.51 ± 4.58	2.932	0.004^a^	0.612
CAMCOG-C	89.87 ± 7.48	83.11 ± 11.04	3.250	0.002^a^	0.678
Orientation	9.62 ± 0.61	9.21 ± 1.18	1.916	0.058^a^	0.400
Language	26.72 ± 2.12	25.34 ± 3.03	2.400	0.018^a^	0.500
Memory	18.86 ± 3.33	17.13 ± 4.31	2.077	0.040^a^	0.433
Attention	6.38 ± 1.01	6.02 ± 1.35	1.364	0.176^a^	0.285
Praxis	11.24 ± 1.23	10.12 ± 1.98	3.065	0.003^a^	0.639
Calculation	2.07 ± 0.23	1.92 ± 0.26	2.782	0.006^a^	0.580
Abstraction	6.41 ± 1.18	5.58 ± 1.74	2.526	0.013^a^	0.527
Perception	8.00 ± 1.03	7.12 ± 1.36	3.365	0.001^a^	0.702
SCWT-A (s)	20.78 ± 7.00	24.62 ± 9.70	−2.071	0.041^a^	−0.432
SCWT-B (s)	23.14 ± 6.69	29.14 ± 8.81	−3.532	0.001^a^	−0.737
SCWT-C (s)	35.08 ± 10.53	42.36 ± 15.77	−2.455	0.016^a^	−0.512
ADL	20.54 ± 1.67	21.16 ± 2.79	−1.208	0.154^a^	−0.252
Mean FD	0.10 ± 0.05	0.10 ± 0.10	0.142	0.887^a^	0.030
WMH			31.637	<0.001^b^	0.577
None to mild, *n* (%)	35(100%)	29(43%)			
Moderate to severe, *n* (%)	0	38(57%)			
LI, *n* (%)	0	16(24%)	9.913	0.002^b^	0.312
EPVS, *n* (%)			31.637	<0.001^b^	0.577
None to mild, *n* (%)	35	49(73%)			
Moderate to severe, *n* (%)	0	18 (27%)			

*BMI, body mass index; CAD, Coronary artery disease; MMSE, Mini-Mental State Examination; MoCA, Montreal Cognitive Assessment; CAMCOG-C, Cambridge Cognitive Examination–Chinese version; SCWT, Stroop Color-Word Test; ADL, activities of daily living scale; FD, framewise displacement; WMH, white matter hyperintensity; LI, lacunar infarction; EPVS, enlarged perivascular space. ^a^two sample T-test, ^b^chi square test*.

**Table 2:** SVD scores for all participants across 3 small vessel disease components.

**SVD scores**	**0**	**1**	**2**	**3**
**Total**, ***n*** **(%)**	35 (34%)	46 (45%)	17 (17%)	4 (4%)

*SVD, small vessel disease*.

### Dynamic Functional Connectivity State Characteristics

**Figure 1:**
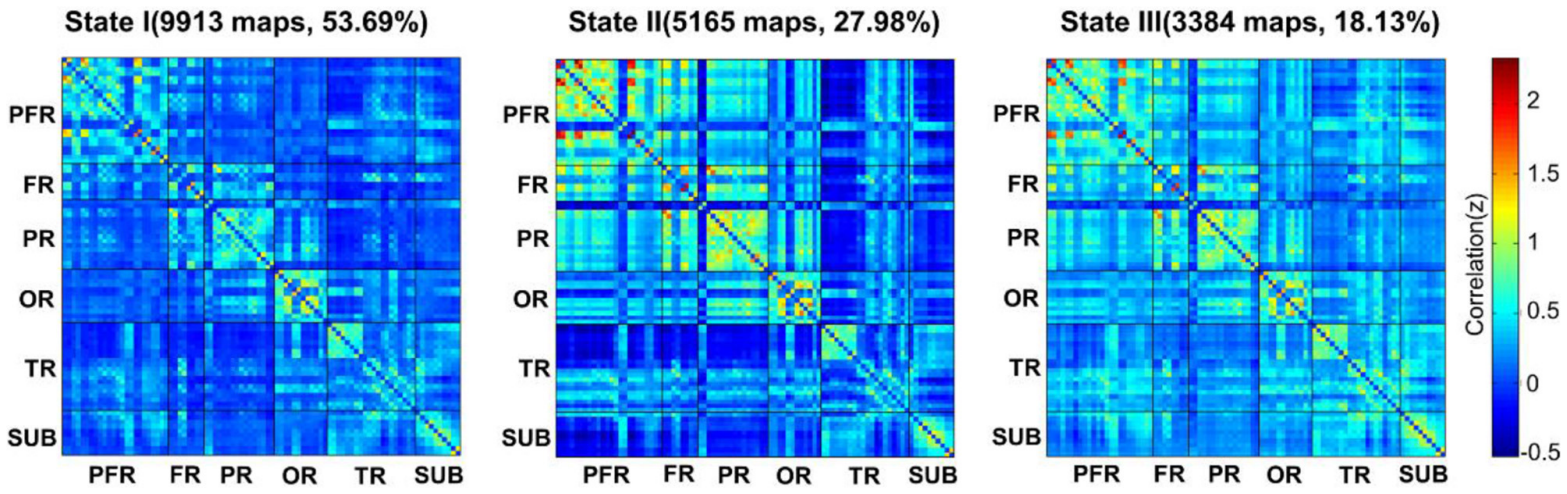
The total numbers and percentages of three state occurrences in all subjects. PFR, prefrontal region; FR, other regions of the frontal lobe; PR, parietal region; OR, occipital region; TR, temporal region; SUB, subcortical region.

Here, three patterns of structured functional connectivity states recurring during individual scans across subjects were identified (Figure 1). Notably, state I (53.69%) was a more frequent and relatively sparse connected state; state II (27.98%) was a less frequent and stronger interconnected state, and state III (18.33%) was an intermediate pattern. As shown in the above three states, state I occurred most frequently.

### Temporal Properties Comparison

**Figure 2:**
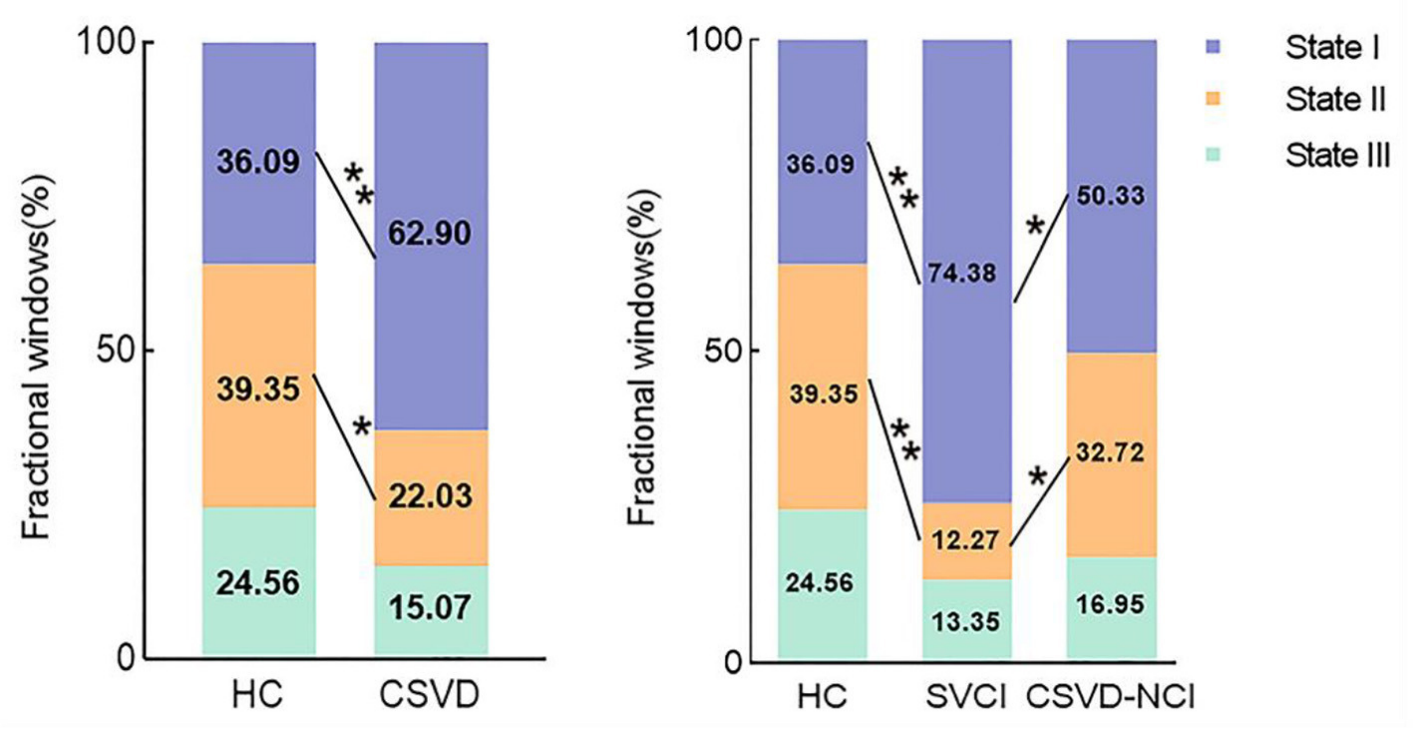
Comparison of the fractional windows in each state between the CSVD and HC groups as well as among the CSVD subgroups by GLM. GLM, general linear model. * *P* < 0.05, ** *P* < 0.01.

As shown in Figure 2 and Supplementary Figure S2, state II (39.35%) was more frequently observed than state I (36.09%) and state III (24.56%) in the HCs, while in the CSVD group, state I occurred much more frequently (62.90%) than state II (22.03%) and state III (15.07%). When comparing the fractional windows after correcting for age, gender, education, and mean FD, state II was less frequent in the CSVD group than in the HC group (*P* = 0.015), and the opposite pattern was observed for state I (*P* = 0.001). Furthermore, state I was more common in the SVCI group (74.38%) than in the CSVD-CU group (50.33%) (*P* = 0.021, Bonferroni correction), while state II was less frequent in the SVCI group (12.27%) than in the CSVD-CU group (32.72%) (*P* = 0.049, Bonferroni correction). In total, the CSVD group exhibited a decreased reoccurrence fraction in state II and an increased reoccurrence fraction in state I compared with those of the HCs.

**Figure 3:**
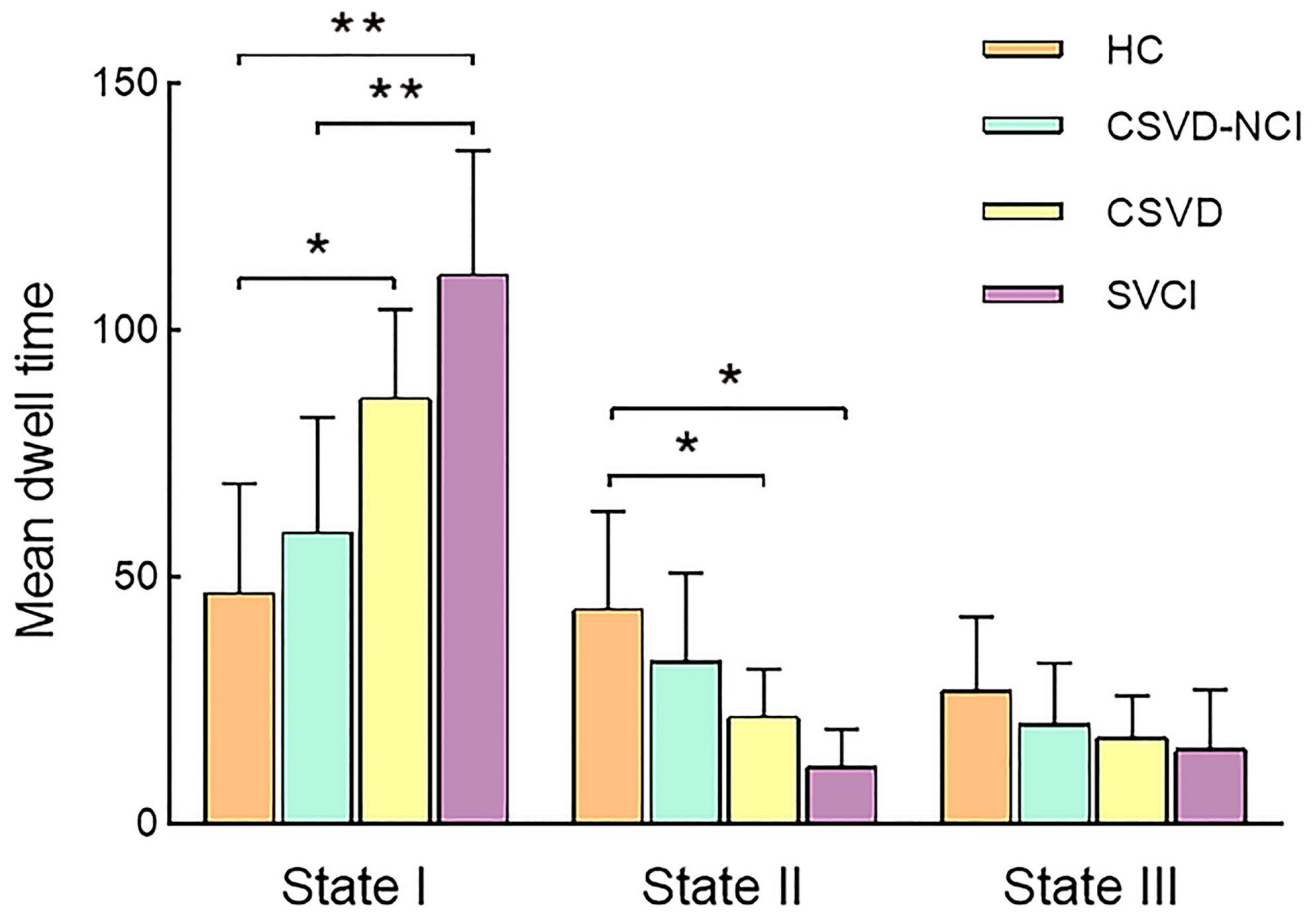
Comparison of the mean dwell time in each state between the CSVD and HC groups as well among the CSVD subgroups by GLM. GLM, general linear model. * *P* < 0.05, ** *P* < 0.01.

Significant differences in the MDT in state I were observed between the CSVD group and HC group after correcting for age, gender, education, and mean FD (*P* = 0.01). The MDT in state II was shorter in the CSVD group than in the HC group (*P* = 0.035). Between-group comparisons among CSVD subgroups revealed that SVCI patients spent significantly more time in state I than CSVD-CU patients (*P* = 0.006, Bonferroni correction) and less time in state II than CSVD-CU patients, but the latter difference was not significant (*P* = 0.216). Overall, these changes in properties showed that CSVD patients, especially SVCI patients, stayed longer in weakly connected state I and dwelled for a shorter period in strongly interconnected state II (Figure 3). The number of transitions was not significantly different between the CSVD group and HC group or among the CSVD subgroups (Supplementary Figures S3, S4).

### Altered DFC Strength Between the CSVD and HC Groups

**Figure 4:**
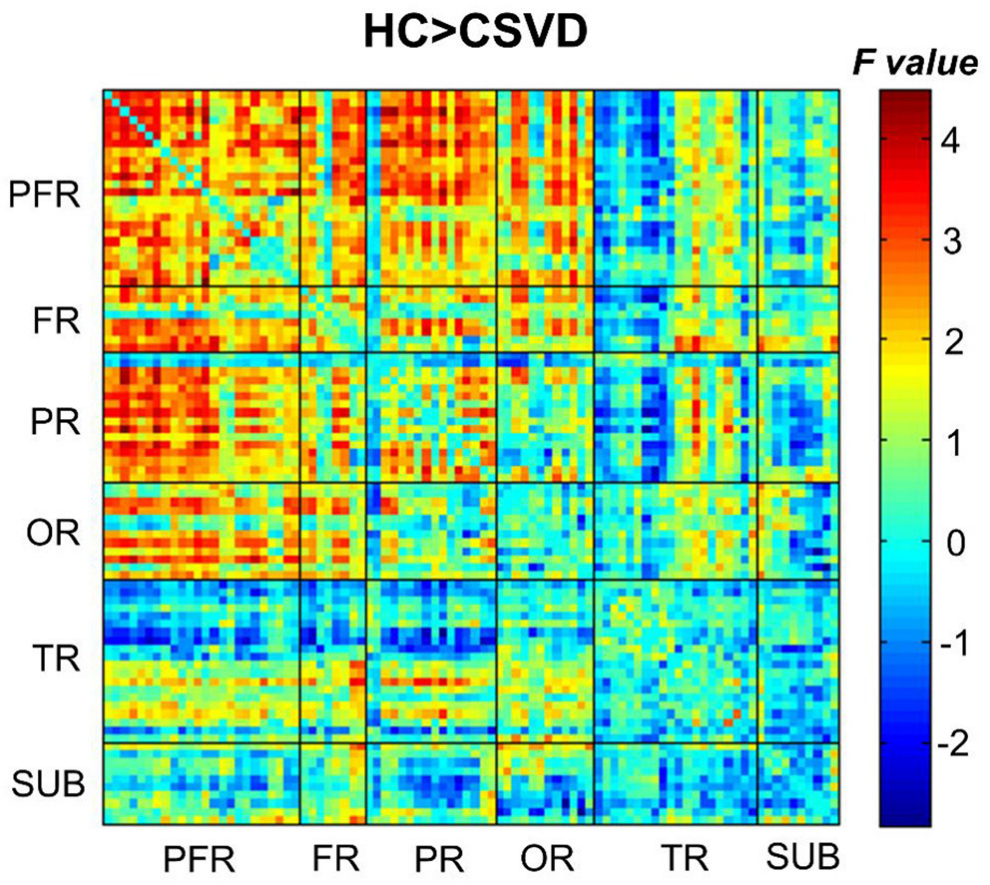
Comparison of the DFC strengths between CSVD patients and HCs (HCs > CSVD) (NBS correction, edge *P* < 0.001, component *P* < 0.05, 1,000 iterations). NBS, network-based statistic.

When comparing the DFC strength in regions between the two groups, the CSVD group showed greatly decreased internetwork and intranetwork functional activity in the frontal lobe, parietal lobe, and occipital lobe (NBS correction) compared to those in the HC groups (Figure 4).

### Validation Analyses

**Figure 5:**
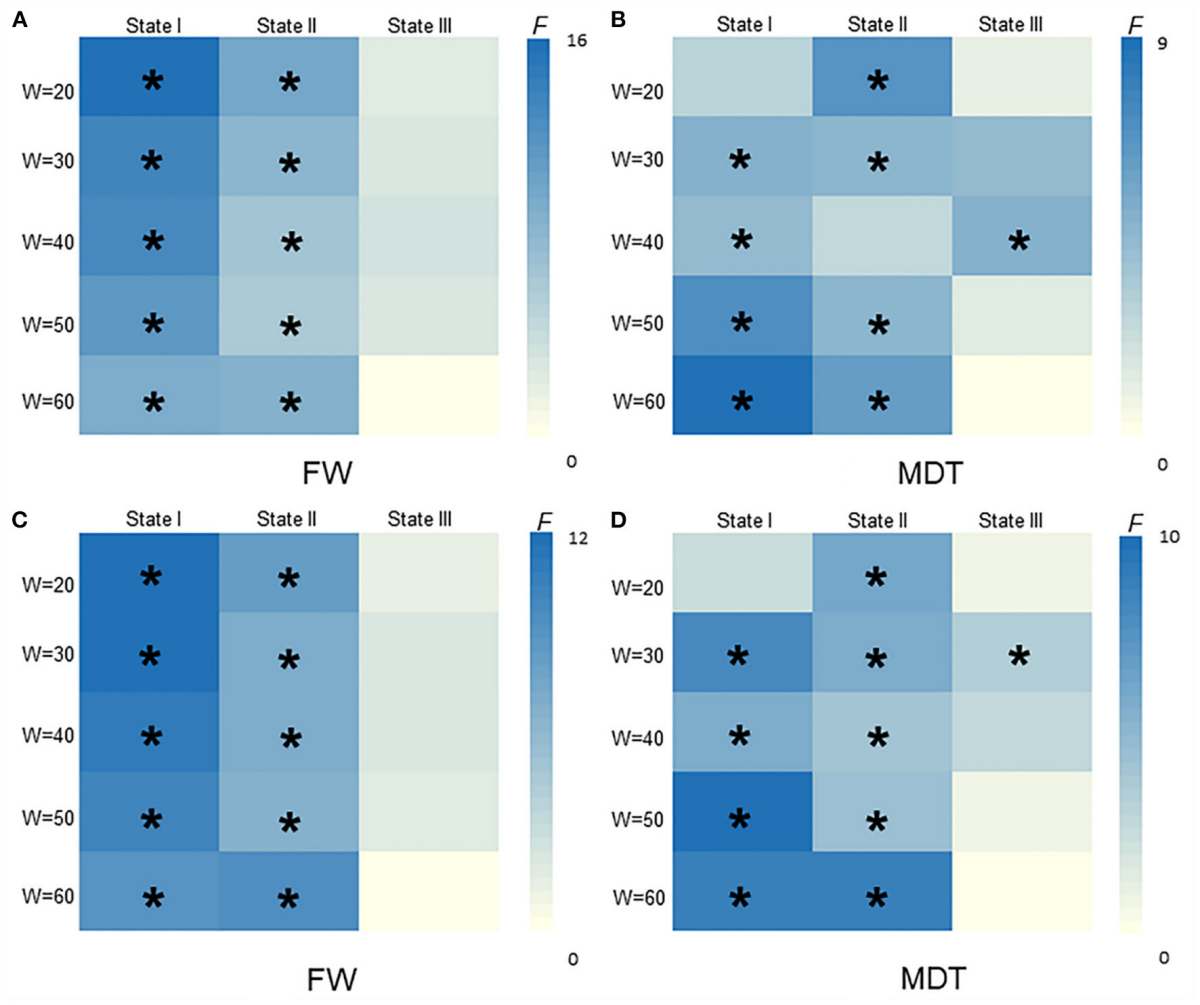
The difference of FW and MDT in different widths of window size among HCs, CSVD-CU, SVCI (20TRs, 30TRs, 40TRs, 50TRs, 60TRs). **(A)** Comparison of FW between HCs and CSVD by GLM. **(B)** Comparison of MDT between HCs and CSVD by GLM. **(C)** Comparison of FW among HCs, CSVD-CU and SVCI by GLM. **(D)** Comparison of MDT among HCs, CSVD-CU and SVCI by GLM. GLM, general linear model; FW, fractional windows; MDT, mean dwell time. * *P* < 0.05.

**Figure 6:**
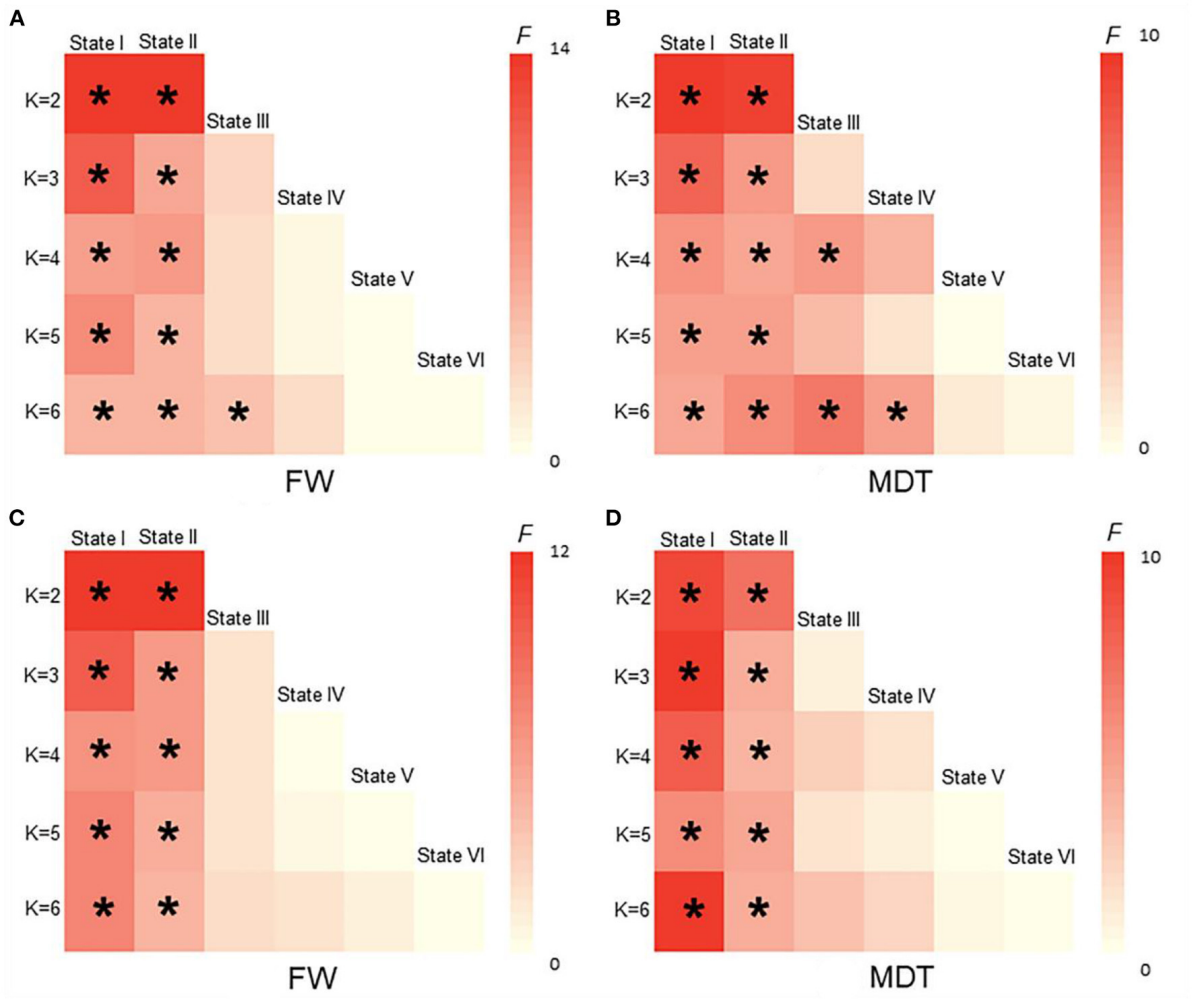
The difference of FW and MDT in different K values among HCs, CSVD, CSVD-CU and SVCI. (*K* = 2, *K* = 3, *K* = 4, *K* = 5, *K* = 6). **(A)** Comparison of FW between HCs and CSVD by GLM. **(B)** Comparison of MDT between HCs and CSVD by GLM. **(C)** Comparison of FW among HCs, CSVD-CU and SVCI by GLM. **(D)** Comparison of MDT among HCs, CSVD-CU and SVCI by GLM. GLM, general linear model; FW, fractional windows; MDT, mean dwell time. * *P* < 0.05.

As shown in Figures 5, 6, the additional sliding window lengths were set at 20 TRs, 30 TRs, 40 TRs, and 60 TRs, and the numbers of clusters were set at 2, 4, 5 and 6. The differences in the fractional windows and MDT in state I and state II between the two groups (CSVD and HCs) and among the three groups (CSVD-CU, SVCI, and HCs) were almost consistent, which indicated the stable results of the DFC analysis.

### Correlation Between Cognition and Temporal Properties

**Figure 7:**
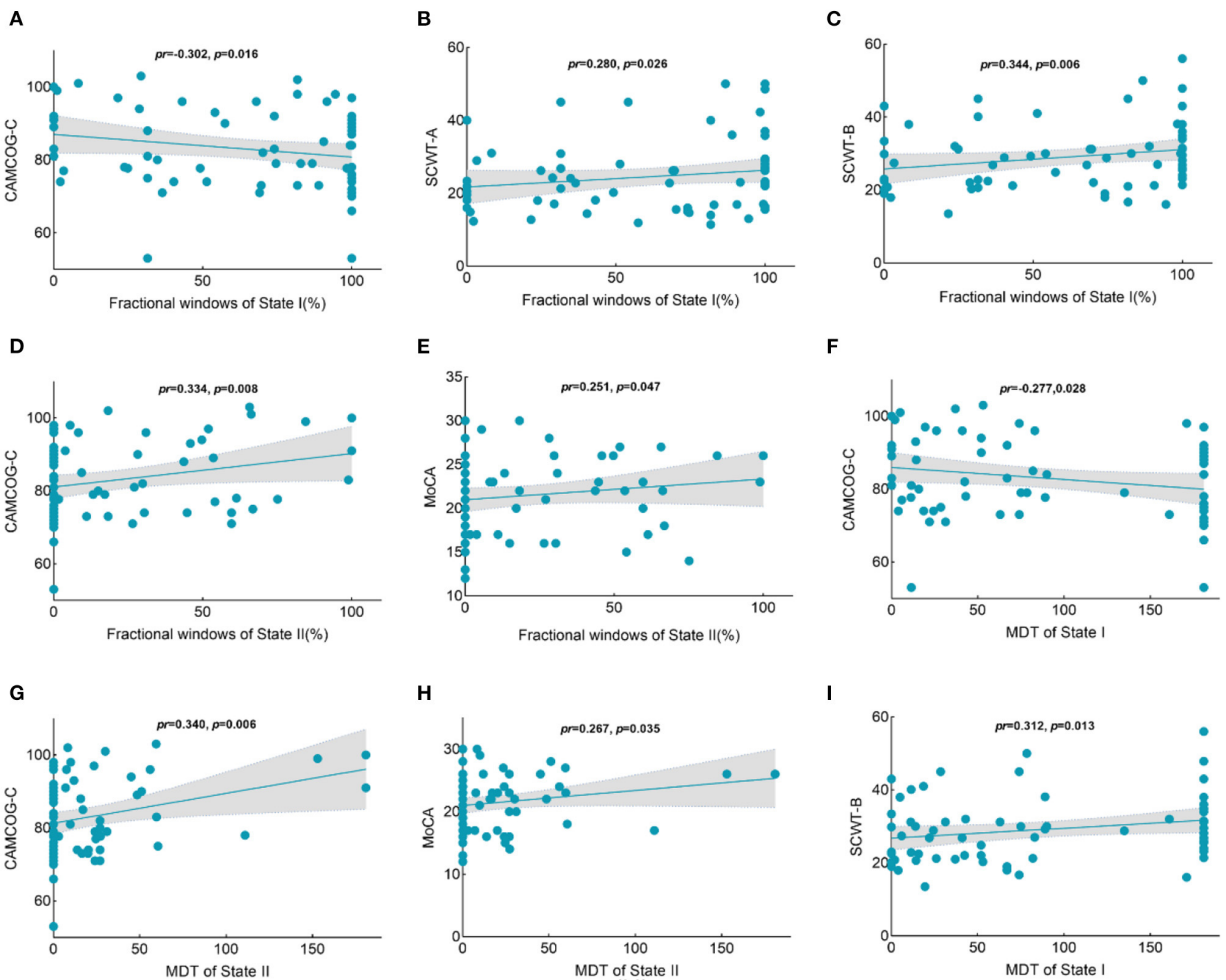
Correlations between the temporal properties with intergroup differences and the cognition scores in the CSVD group. **(A)** A negative relationship was observed between the percentage of state I and the CAMCOG-C scores. **(B)** A positive relationship was observed between the percentage of state I and time consumption in SCWT-A performances. **(C)** A positive relationship was observed between the percentage of state I and time consumption in SCWT-B performances. **(D)** A positive relationship was observed between the percentage of state II and the CAMCOG-C scores. **(E)** A positive relationship was observed between the percentage of state II and MoCA scores. **(F)** A negative relationship was observed between the MDT in state I and CAMCOG-C scores. **(G)** A positive relationship was observed between the MDT in state I and time consumption in SCWT-B performances. **(H)** A positive relationship was observed between the MDT in state II and CAMCOG-C scores. **(I)** A positive relationship was observed between the MDT in state II and MoCA scores. CAMCOG-C, Cambridge Cognitive Examination–Chinese version; SCWT, Stroop Color-Word test; MoCA, Montreal Cognitive Assessment; MDT, mean dwell time.

The fractional windows in state I were negatively related to global cognitive function in the CSVD group (Figure 7A), while the fractional windows in state I were positively associated with time consumption when performing the SCWT-A and SCWT-B tests (Figures 7B,C). Clearly, the results showed that a higher frequency in state I was associated with worse executive function. Moreover, the frequency of state II was positively correlated with global cognitive function (Figures 7D,E). In addition, global cognitive function was negatively related to the MDT in state I (Figure 7F) but positively related to the MDT in state II (Figures 7G,H). Furthermore, a significant positive correlation was shown between the MDT in state I and time consumption when performing the SCWT-B test (Figure 7I).

### Correlation Between SVD Scores and Temporal Properties

Here, no statistical significance was shown between the temporal properties and SVD scores of CSVD group in state I, state II, and state III (*P* > 0.05) (Supplementary Table S1).

## Discussion

The extensively used sliding window approach, combined with straightforward metrics of temporal variability and clustering results for DFC analysis, was utilized. Three states of all subjects were identified by k-means clustering analysis (state I: sparsely connected, state II: strongly connected, state III: intermediate pattern). CSVD patients showed a higher percentage of DFC states in weakly connected state I but a lower percentage of DFC states in strongly connected state II. Moreover, patients with CSVD dwelled significantly longer in state I and for a shorter period in state II than HCs. Notably, these alterations were more significant in the SVCI subgroup. The analysis of DFC strength showed that the CSVD group exhibited substantially decreased internetwork functional activity in the frontal-parietal lobe and frontal-occipital lobe and decreased intranetwork in the frontal lobe and occipital lobe. In addition, a negative correlation was found between cognition and the DFC properties in state I in the CSVD group, while the opposite was found in state II.

### Altered Temporal Properties of Dynamic Functional Connectivity States

In previous research, hypoconnectivity within several large-scale brain networks in CSVD patients compared with HCs has been reported (36, 37). Similarly, a significant hypoconnected pattern in the DFC clustering analysis of CSVD patients was found in this study, especially in the SVCI subgroup. These results indicated that CSVD could transit into the sparse pattern with a higher probability and dwell longer than the HC group. Hypoconnectivity in state I means widespread disruption of the whole brain network, which might result from the disruption of the focal lesion on the traditional imaging or from the cascade effect that spread from the initial focus to remote brain areas (3). Moreover, similar findings have been reported in other neuropsychiatric diseases, such as depression (38, 39), AD (17, 40), and dementia with Lewy bodies (40), as these patients spent more time in the sparsely connected state. Prior studies have revealed the characteristics of disconnection of structural and functional brain networks at a resting state. Therefore, the increased occurrence and longer duration in state I may indicate severe disruption of the brain networks, which leads to an increase in cognitive impairment. The results obtained in this paper could provide more evidence elucidating the disruption mechanism of CSVD from the perspective of DFC.

State II was characterized by highly integrated connections, which showed tightly positive connectivities within regions. When further comparing the DFC properties in the SVCI and CSVD-CU groups, the SVCI group presented an even lower frequency of state II than the CSVD-CU group. The strongly connected state may play a role in cognition processing (41), decreased fractional window and MDT in strongly connected state in CSVD patients suggested that an important connectivity pattern was disrupted in CSVD patients during spontaneous neural activity. Therefore, the lower percentage of DFC occurrence in state II meant lower efficiency in information conveyance, resulting in disruption of integration in specific cognition domains, finally leading to cognitive impairment. The lower frequency of the hyperconnected state implied decreased information transmission and exchange, which was in line with previous studies, thus showing the disconnection of either structural or functional brain networks in CSVD (42–44).

### Altered Average Dynamic Functional Connectivity Strength

The regions with decreased DFC strength were mainly distributed in regions of the frontal lobe, parietal lobe and occipital lobe in CSVD patients, consistent with previous studies (45, 46). In recent decades, disrupted functional activity in the frontal lobe of CSVD patients has been confirmed. The frontal lobe plays pivotal roles in execution and processing and is generally considered to be the initial region involved in the prefrontal-subcortical loop. The frontal-parietal areas are also believed to be associated with several executive control processes related to working memory (45). The decreased DFC strength in the frontal and parietal lobes might be related to reduced flexibility in executive functions, which is in line with a previous study (47). For the occipital lobe, the decreased temporary variability might be associated with impaired visuospatial functions, which have gradually been elucidated to be a vital clinical characteristic (48). Chen et al. reported white matter tract disruption in the inferior frontal-occipital fasciculus in WMH patients, which was in line with our results, indicating the important roles of the occipital lobe in CSVD (49). These results indicated that connectivity impairment in frontal-parietal-occipital areas may be crucial for the pathophysiology of CSVD-related cognitive impairment.

### Relationship Between Temporal Properties and Cognition

Both general cognition and the sub-cognition of executive function were negatively correlated with the proportion of fractional windows and MDT in state I, while positively correlated with fractional windows and MDT in state II. Moreover, better cognitive function was associated with a higher MDT in the strongly connected state II in CSVD. The MDT in hypoconnected state I was negatively related to global cognition, which was characterized by CAMCOG-C scores. Executive function exhibited longer time consumption in SCWT performance. The relevant studies have confirmed hypofunctional connectivity in widespread brain networks in CSVD with cognitive impairment (50, 51). General cognition and executive function were negatively correlated with the occurrence of sparse states, which was consistent with our findings (16). The association of cognitive function with altered dynamic functional connectivity has also been demonstrated in other disease studies (10, 52). These results showed that strongly connected state means more efficient information integration and transmission, thus facilitating the cognition process, whereas sparsely connected states have the opposite effect (10, 41). Here, no significant relationship was found between SVD scores and temporal properties, which indicated that the structural alteration was hard to fully explain the aberrant functional activity, even though the temporal properties were associated with cognition. This study may provide a potential mechanism of CSVD with cognitive impairment from the perspective of dynamic functional connectivity.

### Validation Analyses

In the validation analyses, the results of this work were well replicated when using various analytical parameters, including different parameters of sliding window lengths and k-means clusters. With the number of clusters increasing, the brain variability between the groups was still readily apparent. These observations indicated that the abnormal temporal features and network topological properties in patients with CSVD remained stable, indicating that these parameters can be used as neuroimaging markers to distinguish patients with CSVD from healthy people as well as to distinguish SVCI patients from CSVD-CU patients. Herein, the findings validate the results obtained by DFC, which were consistent with previous studies and further confirmed the reliability and repeatability of DFC analysis (53, 54).

### Limitations

However, the current research still has several limitations. First, the specific differences in DFC according to the different imaging features of CSVD or the severity of cognitive impairment were not investigated due to the relatively small sample size. Moreover, none of the participants underwent susceptibility-weighted imaging or T2^*^ imaging; therefore, the cerebral microbleed information remains unclear. In addition, the widely used sliding-window approach was adopted to extract the dynamics of functional connectivity in the current study. Furthermore, these results should be validated by other methods, such as Bayesian and frequentist methods, in the future. Finally, longitudinal and large sample studies are required to confirm the alteration of DFC in CSVD and its relationship with cognitive impairment.

## Conclusion

In this study, the temporal property alterations of functional connectivity in CSVD and cognitive impairment analyses were emphatically investigated from the perspective of DFC. The patients with CSVD spent more time in a sparsely connected state and less time in a strongly connected state in all reoccurring states, which might have been associated with cognitive impairment. All the results herein could facilitate a deeper understanding of the potential neural mechanisms of cognitive impairment in CSVD.

## Data Availability Statement

The raw data supporting the conclusions of this article will be made available by the authors, without undue reservation.

## Ethics Statement

The present study was approved by the First Affiliated Hospital of Anhui Medical University Subcommittee on Human Studies (Ethics Ref. Quick-PJ2021-15-33). The patients/participants provided their written informed consent to participate in this study.

## Author Contributions

WY and XZho: conceptualization, methodology, data analysis, visualization, and writing of the original draft. CL, MY, KW, WZha, WZhu, and ML: investigation, execution, data collection, and revision of the manuscript. ZS, YQ, and XZhu: conceptualization, funding acquisition, and revision of the manuscript. All authors read and approved the final version of the manuscript.

## Funding

This work was supported by Key Research and Development Projects of Anhui Province (202104j07020031), the Natural Science Foundation of Anhui Province (1908085QH322), the Basic and Clinical Cooperative Research Promotion Plan of Anhui Medical University (2020xkjT026), and the National Natural Science Foundation of China (81771154).

## Conflict of Interest

The authors declare that the research was conducted in the absence of any commercial or financial relationships that could be construed as a potential conflict of interest.

## Publisher's Note

All claims expressed in this article are solely those of the authors and do not necessarily represent those of their affiliated organizations, or those of the publisher, the editors and the reviewers. Any product that may be evaluated in this article, or claim that may be made by its manufacturer, is not guaranteed or endorsed by the publisher.
