# Supplementary material for: The Clustering Analysis of Time Properties in Patients With Cerebral Small Vessel Disease: A Dynamic Connectivity Study
Source: Front Neurol. 2022 Jun 20;13:913241. doi: 10.3389/fneur.2022.913241 (PMC9251301; doi:10.3389/fneur.2022.913241)
Supplement: Supplementary file 1 [file Table_1.docx]

**Supplementary Materials for**

**the clustering analysis of time properties in patients with cerebral small vessel disease: a dynamic connectivity study**

Wenwen Yin^1,†^**^,^** Xia Zhou^1,†^**,** Chenchen Li^1^**,** Mengzhe You^1^**,** Ke Wan^1^**,** Wei Zhang^1^**,** Wenhao Zhu^1^**,** Mingxu Li^1^**,** Xiaoqun Zhu^1^**,** Yinfeng Qian^2^**,** Zhongwu Sun^1,*^

^1^Department of Neurology, The First Affiliated Hospital of Anhui Medical University, Hefei, China

^2^Department of Radiology, The First Affiliated Hospital of Anhui Medical University, Hefei, China

† These authors have contributed equally to this work and share first authorship.

^*^**Corresponding author**

Zhongwu Sun MD., Ph.D. Professor of Neurology

Department of Neurology, the First Affiliated Hospital of Anhui Medical University, 218 Jixi Road, Hefei, Anhui Province 230022

Tel: 86-10-62922328

Email: sunzhwu@126.com

**This file includes: Figure S1-S4, Table S1**

**
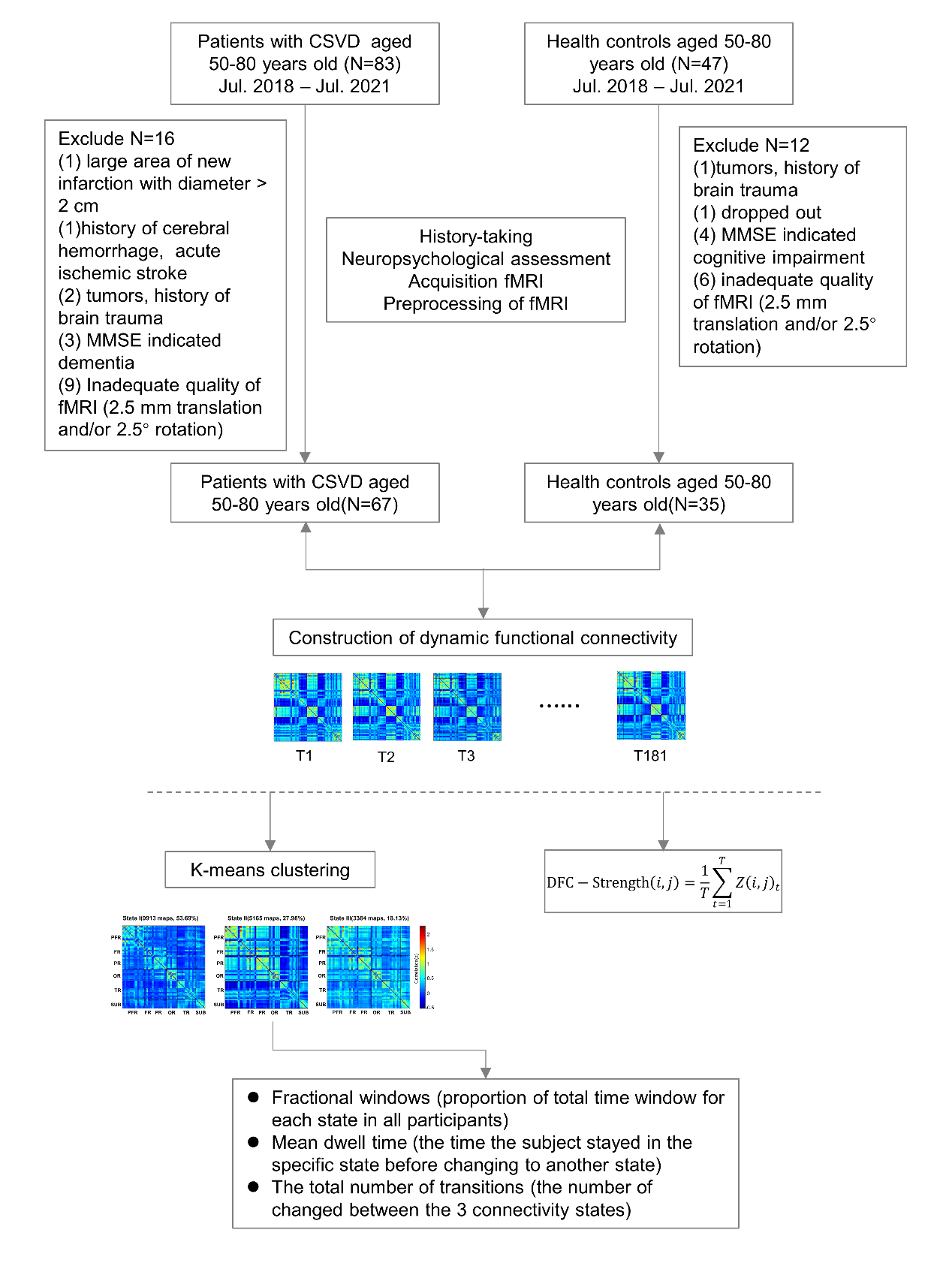
**

**Figure S1** Flowchart of inclusion and exclusion criteria for participants and data processing in the study.


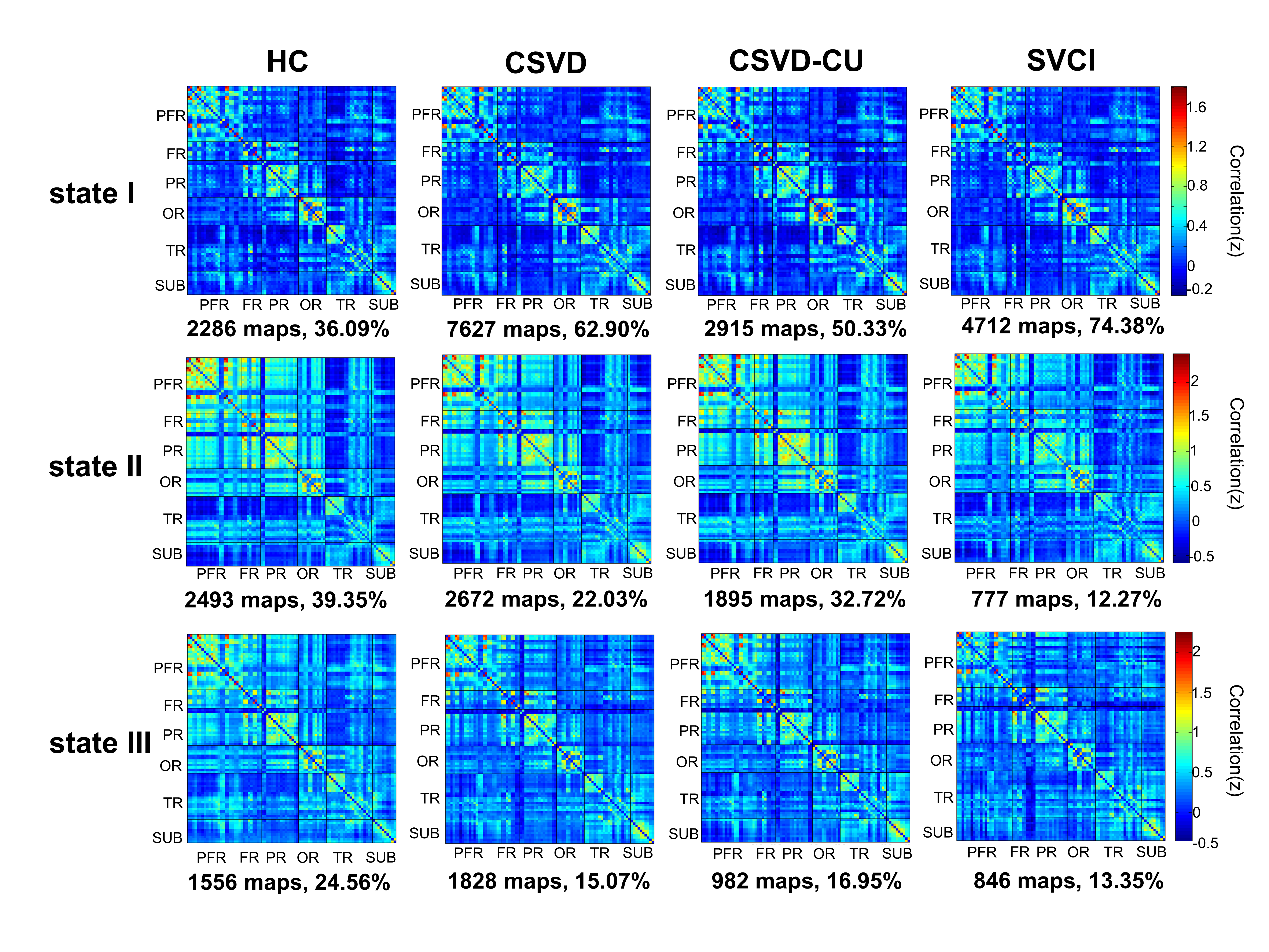


**Figure S2** Functional connectivity state distribution in each group and subgroups (HCs, CSVD, CSVD-CU, and SVCI group). HCs: healthy controls; CSVD-CU: cerebral small vessel disease with cognitively unimpaired; SVCI: subcortical vascular cognitive impairment.


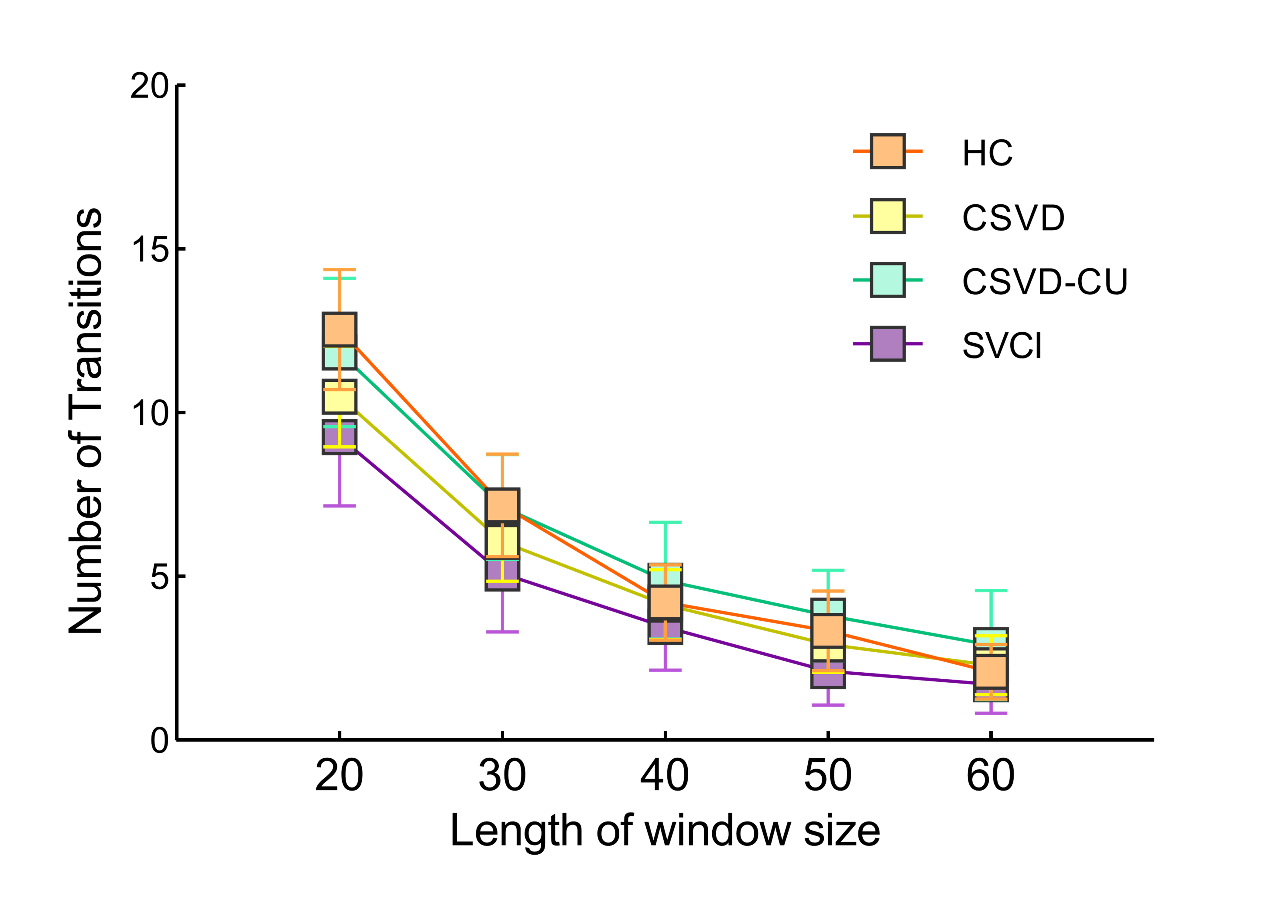


**Figure S3** Numbers of transitions changes at different widths of window size in different groups.


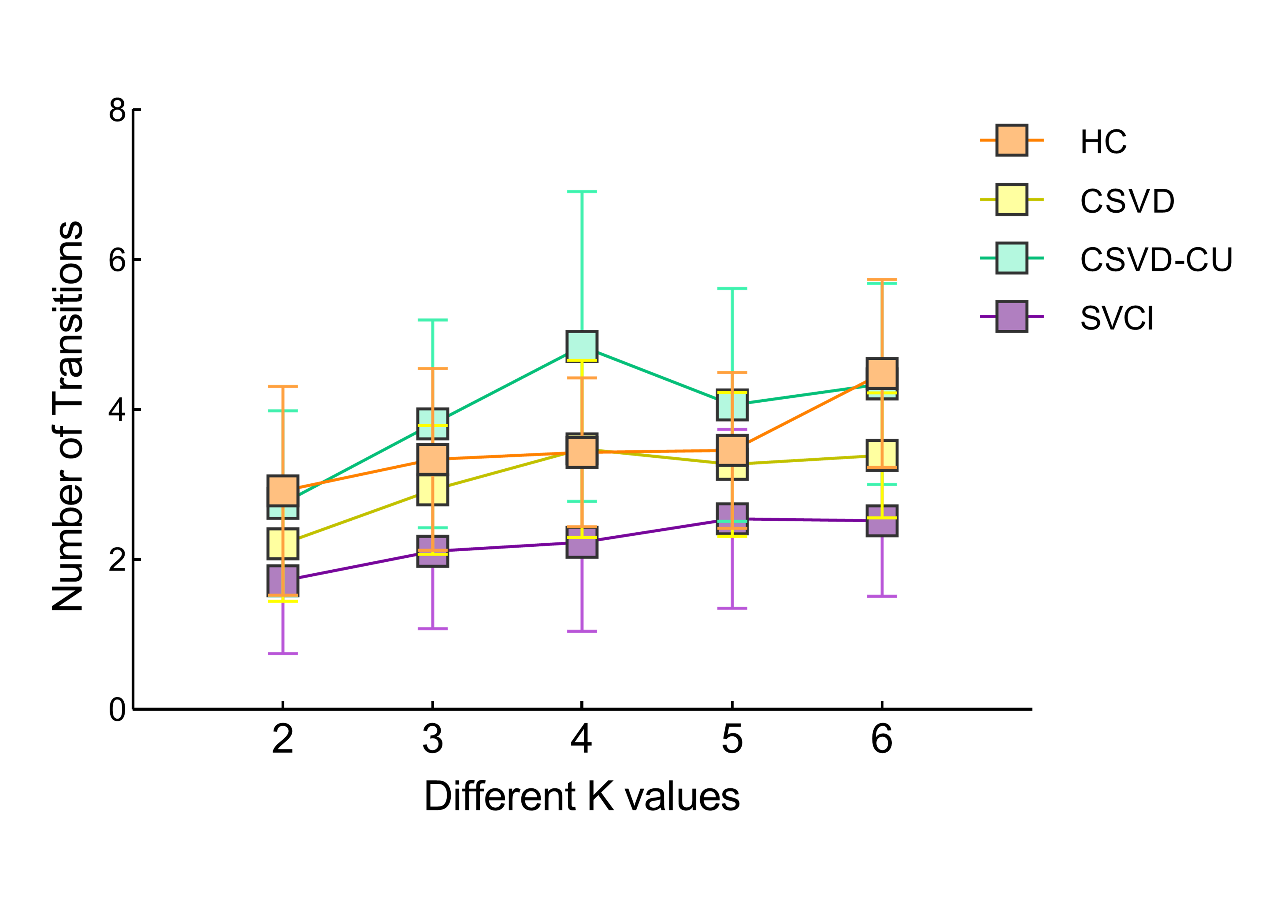


**Figure S4** Numbers of transitions changes at different K values in different groups.

**Table S1: The association between SVD scores and temporal properties in CSVD group.**

|  | **r** | ***P*** |
| --- | --- | --- |
| **FW of state I** | -0.13 | 0.295 |
| **FW of state II** | 0.143 | 0.247 |
| **FW of state III** | 0.038 | 0.757 |
| **MDT of state I** | -0.115 | 0.354 |
| **MDT of state II** | 0.147 | 0.236 |
| **MDT of state III** | 0.043 | 0.727 |
| **NT** | 0.031 | 0.802 |

FW: fractional windows, MDT: mean dwell time, NT: number of transitions
